# Elevated Serum Levels of Cysteine and Tyrosine: Early Biomarkers in Asymptomatic Adults at Increased Risk of Developing Metabolic Syndrome

**DOI:** 10.1155/2015/418681

**Published:** 2015-03-04

**Authors:** Nina Mohorko, Ana Petelin, Mihaela Jurdana, Gianni Biolo, Zala Jenko-Pražnikar

**Affiliations:** ^1^Science and Research Centre, University of Primorska, Garibaldijeva 1, SI-6000 Koper, Slovenia; ^2^Faculty of Health Sciences, University of Primorska, Polje 42, SI-6310 Izola, Slovenia; ^3^Department of Medical, Surgical and Health Sciences, Division of Internal Medicine, AOUTS, University of Trieste, Strada di Fiume 447, 34149 Trieste, Italy; ^4^Clinica Medica, Ospedale di Cattinara, Strada di Fiume 149, 34149 Trieste, Italy

## Abstract

As there is effective intervention for delaying or preventing metabolic diseases, which are often present for years before becoming clinically apparent, novel biomarkers that would mark metabolic complications before the onset of metabolic disease should be identified. We investigated the role of fasting serum amino acids and their associations with inflammatory markers, adipokines, and metabolic syndrome (MetS) components in subjects prior to the onset of insulin resistance (IR). Anthropometric measurements, food records, adipokines, biochemical markers, and serum levels of amino acids were determined in 96 asymptomatic subjects aged 25–49 years divided into three groups according to the number of MetS components present. Cysteine and tyrosine were significantly higher already in group with one component of MetS present compared to subjects without MetS components. Serum amino acid levels correlated with markers of inflammation and adipokines. Alanine and glycine explained 10% of insulin resistance variability. The role of tyrosine and cysteine, that were higher already with 1 component of MetS present, should be further investigated as they might point to future insulin disturbances.

## 1. Introduction

While people are becoming increasingly less active and more obese, the incidence of metabolic syndrome (MetS) is growing at an alarming rate [1]. MetS is a cluster of metabolic risk factors, including central adiposity, hyperglycemia, hypertension, and dyslipidemia that occur simultaneously in the same individual [2, 3]. Its underlying mechanisms remain only partly understood. However, a strong consensus has been built on the role of abdominal obesity and insulin resistance (IR) as pathogenic factors potentially leading to all defining alteration and to the further development of metabolic diseases [4]. In addition, comparison of obese and lean subjects has evoked additional hypotheses to explain the pathophysiological pathways of obesity associated metabolic disorders including systemic low-grade inflammation due to changes in circulating inflammatory cytokines and adipokines [5, 6] and intestinal microbiota composition [7].

Metabolic diseases (e.g., diabetes) are often present for years before becoming clinically apparent. At present, clinical and laboratory predictors such as body mass index or fasting glucose can be helpful in determining diabetes risk [8], but they often reflect disease, which is already present. In addition, IR and type 2 diabetes mellitus (T2DM) might remain broadly undiagnosed in overweight and obese subjects with normal blood glucose concentration as they are conditions of a broadly dysfunctional metabolic physiology that in addition to glucose metabolism involve considerable changes in amino acid and fat metabolism [9].

Given the availability of effective intervention for delaying or preventing metabolic diseases [10] and given the increasing prevalence of obesity worldwide, it remains a challenge to identify asymptomatic (overweight and obese) individuals who are at increased risk of developing MetS. Biomarkers for MetS are potential tools to identify such individuals and beside IR, several others, including inflammation markers, markers of lipid peroxidation, adipokines, markers of uric acids, and some others, have been found to predict the risk of MetS [4, 11]. Due to comprehensive metabolic profiling studies, recently, amino acids have been proposed as new biomarkers reflecting metabolic signatures of insulin action in obese individuals [12–14]. Moreover, a positive relationship between branched-chain and aromatic amino acids and risk for future diabetes and IR was demonstrated [15, 16] and it was found that branched-chain, aromatic amino acids and orosomucoid were associated with MetS and could be relevant biomarkers of obesity associated cardiometabolic disorders [17].

The linkage between some amino acids, obesity, and IR is well recognized. Moreover, few studies have also evaluated adipokines and inflammatory cytokines for their sensitivity to reflect MetS components. However, the relation between serum amino acids, adipokines, inflammatory cytokines, and components of MetS in various states of obesity, including progression from the lean to obese state, prior to the onset of IR, remains incompletely understood. Therefore, the aim of the present study was to analyze the associations between specific amino acids and experimental and clinical measures such as body shape index (ABSI), blood lipids, inflammatory markers, and adipokines, which have been confirmed to be risk factors for MetS, in lean and obese individuals prior to the onset of IR.

## 2. Patients and Methods

### 2.1. Participant Recruitment and Characteristics

A population of one hundred eighty-two (70 males and 112 females) adults, local residents between 25 and 49 years of age, were enrolled in this cross-sectional study. Subject sampling, recruitment, and evaluation have been described earlier [18]. Participants were included if they had BMI between 20 and 34.9, did not take any medications for lipid disorders or anti-inflammatory drugs, did not have any cardiovascular, endocrine, acute, or chronic inflammatory disease, T2DM, reported stable weight over the last three months, completed the questionnaires, and signed an informed consent form approved by the Slovenian National Medical Ethics Committee (No. 56/08/11 bis). Ninety-six subjects who met the inclusion criteria were split into three different groups according to the presence and the number of components of MetS. MetS was evaluated according to the Harmonization definition: hypertriglyceridemia (≥1.7 mmol/L), HDL (<1 mmol/L in men and <1.3 mmol/L in women), large WC (≥94 cm in men and ≥80 cm in women), elevated blood pressure (systolic ≥ 130 mmHg and/or diastolic ≥ 85 mmHg), and elevated plasma glucose (≥5.6 mmol/L) [3]. MetS0 group consisted of subjects without any component of MetS, in MetS1 group subjects were having one component of MetS, and in MetS2 group subjects were with 2 and more components of MetS.

### 2.2. Anthropometric Measurements and Measurement of Resting Metabolic Rate

Anthropometric measurements were obtained using standard protocols and techniques after an overnight fast by the same, trained, investigator. Subject height was measured using a Leicester Height Measure (Invicta Plastics Limited, Oadby, England), with subjects standing wearing light clothes and no shoes. Weight and height were measured to the nearest 0.1 kg and 0.1 cm precision, respectively. Waist circumference (WC) was measured at the midpoint between the inferior costal margin and the superior border of the iliac crest on the midaxillary line, whereas the hip circumference (HC) was measured as the greatest circumference around the buttocks. BMI was defined as weight (kg) divided by height squared (m^2^). Bioelectrical impedance analysis (BIA) Tanita BC 418MA (Tanita Corporation, Arlington Heights, IL) was used to analyze the total body fat and body composition. Obtained data was analyzed with the provided software. To avoid the drawbacks regarding the direct relationship between BMI and WC, ABSI was calculated according to the following formula: ABSI = WC/(BMI^2/3^ · height^1/2^), with WC and height expressed in meters and weight in kilograms [19].

In addition, resting metabolic rate (RMR) was determined using a hand-held indirect calorimeter MedGem (Medical Home Solutions, Inc., Golden, CO). All RMR measurements were performed after an overnight fast in a quiet thermoneutral environment (20–22°C).

### 2.3. Dietary Assessment and Physical Fitness Testing

To assess the energy intake, subjects were asked to write down a three-day weighed food record for three consecutive days (including one weekend day). Subjects were fully briefed on how to complete the diaries by a trained dietitian. Subjects were asked to continue their normal diet. We asked them to include food labels and recipes for mixed dishes in their record. Dietary records were analyzed using an online, freely assessable, dietary assessment and planning tool for the analysis of a food diary, named Open Platform for Clinical Nutrition (OPEN), (http://www.opkp.si/en_GB/cms/vstopna-stran). OPEN food composition data was taken from the Slovenian food composition database (FCDB) [20] or if not available here from the Souci-Fachmann-Kraut FCDB [21] and/or from the USDA National Nutrient Database for Standard Reference (http://www.ars.usda.gov/Services/docs.htm?docid=8964). Food composition data applied by the OPEN met the European standard for food data CEN/TC 387, available at http://www.cen.eu/. In addition, assessment of aerobic capabilities for each subject was determined by physical fitness. The participants had to walk (brisk walking) over 2 km and fitness index (FI), based on age and BMI, was calculated by scores of walking time and pulse rate measured at the cervical aorta. Mentioned program was developed by the UKK Institute for Health Promotion Research, Tampere, Finland [22].

### 2.4. Biochemical Measurements

After an overnight fasting, 6 mL of venous blood was withdrawn by a trained nurse. Blood samples for biochemical and hormonal determinations were collected in tubes for serum separation. Serum was separated by centrifugation with coagulated blood at 2000 rpm for 10 min at 4°C. All samples were then frozen and stored at −20°C until subsequent analysis. Enzyme-linked immunosorbent assays (commercially available kits) were used for measuring serum levels of adiponectin, visfatin, interleukin 6 (IL-6), resistin, and tumor necrosis factor *α* (TNF-*α*). Assay sensitivity was 30 pg/mL for visfatin, 10 pg/mL for adiponectin, <1 pg/mL for IL-6, and <2 pg/mL for TNF-*α*. Assays interassay and intra-assay CVs were typically <10%. Fasting serum glucose, C-reactive protein (CRP), total cholesterol, low density lipoprotein- (LDL-) cholesterol, high density lipoprotein- (HDL-) cholesterol, and triacylglycerols were determined using an AU 680 analyzer (Beckman Coulter) and Olympus reagents. In addition, a 2000 iSR analyzer (Abbott Architect) and Abbott reagents were used for measuring serum levels of insulin. The estimation of IR was done using the well-established homeostasis model assessment (HOMA) formula [23].

Serum levels of 10 amino acids (cysteine (Cys), alanine (Ala), glycine (Gly), leucine (Leu), methionine (Met), phenylalanine (Phe), proline (Pro), serine (Ser), threonine (Thr), and tyrosine (Tyr)) were analyzed by gas chromatography-mass spectrometry (GC-MS), as previously described [24]. To sum up, known amounts of ^2^H_2_-cysteine, ^15^N-alanine, ^15^N-glycine, ^13^C-leucine, [1-^13^C, methyl-^2^H_3_]methionine, ^13^C-phenylalanine, ^15^N-proline, ^15^N-serine, ^15^N-threonine, and ^2^H_2_-tyrosine (Cambridge Isotope Laboratories) were used as internal standards. Serum samples (200 *μ*L) with a known amount of added internal standard were treated with sulphosalicylic acid (SSA; 200 *μ*L, 10%) and then centrifuged and purified on a cationic resin (AG50W-X8; Bio-Rad, Hercules, CA). After that, samples were lyophilized and amino acids were derivatized by the addition of 50 *μ*L acetonitrile and 50 *μ*L MTBSTFA (N-methyl-N-(*tert*-butyldimethylsilyl)-trifluoroacetamide) and by heating at 90°C for 45 min. After derivatization, samples were injected into a GC-MS system (HP 5890, Agilent Technologies, Santa Clara, CA, USA). Serum concentrations of amino acids were monitored once; each sample had its own internal standard, as follows:* m/z*: Cys* m/z* 406/408, Ala* m/z* 158/159, Gly* m/z* 218/219, Leu* m/z* 302/303, Met* m/z* 320/324, Phe* m/z* 336/337, Pro* m/z* 184/185, Ser* m/z* 362/363, Thr* m/z* 404/405, and Tyr* m/z* 466/468.

### 2.5. Statistical Analysis

Variables are presented as means ± SD. Descriptive statistics were tested before statistical analysis as being normally distributed; data that was not normally distributed (serum levels of LDL cholesterol, triacylglycerols, CRP, insulin, HOMA-IR, visfatin, and TNF-*α*,) was logarithmically transformed for subsequent analysis. To investigate the metabolic profile in subjects with or without component of MetS, subjects were split into three different groups according to the presence and the number of components of MetS (MetS0 group consisted of subjects without any component of MetS, in MetS1 group subjects were having one component of MetS, and in MetS2 group subjects were with 2 and more components of MetS). Anthropometrical, nutritional, and physical characteristics and serum metabolites were compared between MetS groups using an independent-sample *t*-test. Pearson's correlation analyses (crude and adjusted for age, gender, protein intake, fitness index, and RMR) were performed to detect different associations between serum amino acids and components of MetS and/or markers of inflammation. In addition, hierarchical multiple regression analysis was used to identify predictors of IR score. SPSS software (IBM SPSS version 19.0, Chicago, IL) was used for all analyses. A *P* value of less than 0.05 was considered statistically significant.

## 3. Results

### 3.1. Baseline Characteristics and Physiologic Measures

Ninety-six subjects underwent baseline evaluation. They were split into three different groups according to the presence and the number of components of MetS. MetS0 group consisted of subjects without any component of MetS, in MetS1 group subjects were having one component of MetS and in MetS2 group subjects were with 2 and more components of MetS. MetS0 group comprised 65% women and MetS1 comprised 69%, whereas MetS2 comprised 67%. Mean age of subjects in group MetS2 was 39.5 ± 6.2 years, and their mean BMI was 29.0 ± 3.5 kg/m^2^, compared to 36.7 ± 5.6 years and 26.4 ± 4.2 kg/m^2^ for the subjects in group MetS1 and to 36.6 ± 6.5 years and 21.6 ± 2.2 kg/m^2^ for the subjects in group MetS0. Due to the heterogeneity of BMI between three groups and because of the direct relationship between BMI and WC, ABSI is added in Table 1. Additional clinical data are also provided in Table 1.

Based on 3-day food record, subjects in group MetS2 had a higher dietary intake of protein (*P* = 0.049) and a trend toward an increase in saturated fat consumption. Physical activity measured by fitness index was significantly lower in subjects with components of MetS (MetS1 and MetS2). However, there was no difference in resting metabolic rate between studied groups.

Subjects in groups MetS1 and MetS2 had a 37% and 63% increase in fat mass compared to controls in group MetS0. Subjects, who had 3 and more (*n* = 11) components of MetS, were also less sensitive to glucose than MetS0 (data not shown). Indeed, the HOMA-IR index was 2-fold higher in subjects in group MetS2 than in controls (*P* < 0.001). IR was diagnosed in 5 subjects from MetS2 (HOMA-IR > 3.60 if BMI > 27.5 kg/m^2^ [25]), but mean HOMA-IR was below the threshold.

### 3.2. Hormones, Cytokines, and Metabolites

As shown in Table 2, the adipocyte-derived hormones adiponectin and visfatin were reciprocally altered by presence of components of MetS, such that visfatin levels were 3-fold higher in subjects in group MetS2 (*P* < 0.001), whereas adiponectin levels were 43% lower (*P* = 0.014). Resistin levels were not significantly different in subjects in groups MetS1 and MetS2 versus subjects in group MetS0. Among the proinflammatory and anti-inflammatory cytokines measured, TNF-*α* and CRP differed between three studied groups. Furthermore, CRP levels were 3-fold and 4.5-fold higher in group MetS1 and group MetS2, respectively. Mean fasting blood glucose, insulin, total cholesterol, LDL-cholesterol, and triacylglycerols levels were higher in group MetS2 compared to group MetS0. On the other hand, HDL-cholesterol and bilirubin levels were lower in group MetS2, compared to group MetS1 and MetS0. We measured serum amino acids by tandem mass spectrometry. Among a total of 10 amino acids measured, 3 were not different in subjects having 1, 2, or more components of MetS versus control subjects (Thr, Pro, and Met). Five other amino acids were elevated in subjects having components of MetS versus controls (Ala, Leu, Phe, Tyr, and Cys), whereas Gly and Ser levels were lower in these subjects. Cys and Tyr were significantly higher already in MetS1 compared to MetS0 (Table 2).

### 3.3. Evaluation of the Associations between Amino Acids and Components of MetS

Changes in blood concentrations of Ala, Gly, Leu, Cys, Tyr, and Phe were apparent with obesity and IR.

### 3.4. Associations between Sulfur Amino Acids and Components of MetS and Markers of Inflammation

Evaluation of the associations between Cys and Met with parameters of body composition reveals significant strong associations between Cys and BMI, body fat, and WC, as observed in Tables 3 and 4. Furthermore, even when adjusting for age, gender, protein intake, RMR, and FI, significant associations remained between Cys and HOMA (Table 3; *r* = 0.443, *P* = 0.001), CRP (Table 3; *r* = 0.378, *P* = 0.003), WC (Table 3; *r* = 0.512, *P* < 0.001), ABSI (Table 3; *r* = 0.323, *P* = 0.002), TNF-*α* (Table 3; *r* = 0.259, *P* = 0.038), and HDL-cholesterol (Table 3; *r* = −0.281, *P* = 0.032). On the other hand, we did not observed any significant association between serum levels of Met and components of MetS nor for Met metabolite, homocysteine (data not shown).

### 3.5. Associations between Tyr and Phe and Components of MetS and Markers of Inflammation

**Figure 1:**
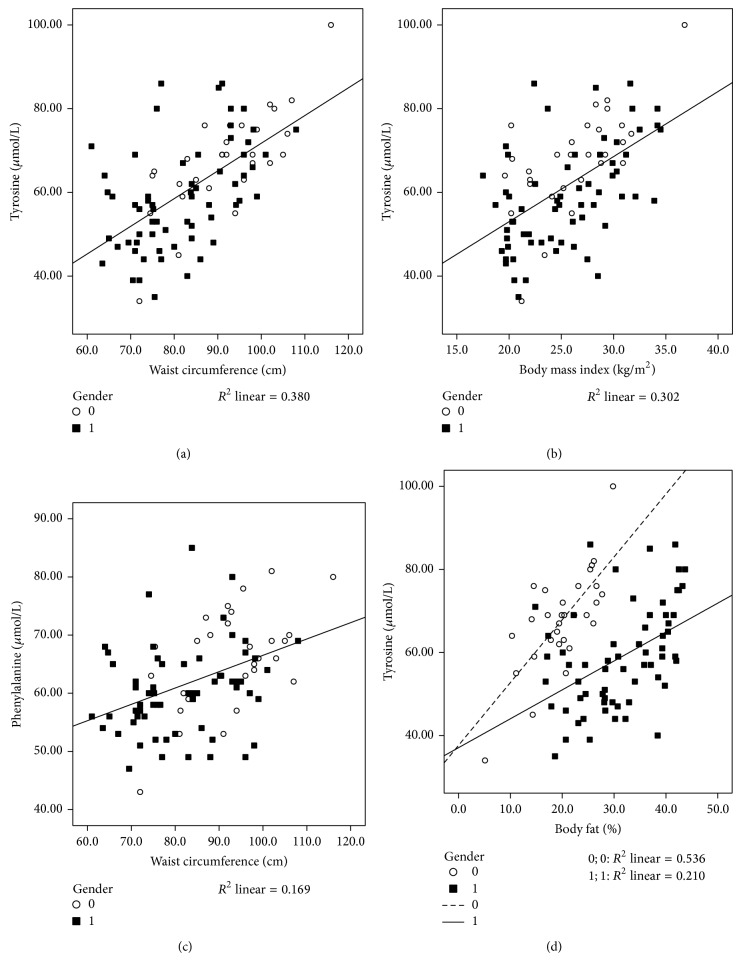
*Associations between aromatic amino acids and components of MetS*. Evaluation of the associations between tyrosine with components of MetS reveals significant strong associations between tyrosine and waist circumference (*r* = 0.621, *R* ^2^ = 0.380; *P* < 0.001) (a), body mass index (*r* = 0.550, *R* ^2^ = 0.302; *P* < 0.001) (b), and % of body fat (*r* = 0.733, *R* ^2^ = 0.536; *P* < 0.001 for male subjects; *r* = 0.458, *R* ^2^ = 0.210; *P* = 0.002 for female subjects) (d). A significant strong association between phenylalanine and waist circumference is evident in (c) (*r* = 0.411, *R* ^2^ = 0.169; *P* < 0.001). ○, male subjects; ■, female subjects. Associations were analyzed by Pearson's correlation analyses (crude). A *P* value of less than 0.05 was taken as a statistically significant difference between the tested parameters.

Evaluation of the associations between Tyr with components of MetS reveals significant strong associations between Tyr and WC, body fat, SBP, glucose, triacylglycerols, HDL-cholesterol, CRP, HOMA, adiponectin, and TNF-*α*, as observed in Table 3 and Figure 1. Furthermore, even when adjusting for age, gender, protein intake, RMR, and FI, significant associations remained between Tyr and HOMA (Table 3; *r* = 0.313, *P* = 0.020), CRP (Table 3; *r* = 0.293, *P* = 0.029), WC (Table 3; *r* = 0.5040, *P* < 0.001), ABSI (Table 3; *r* = 0.273, *P* = 0.009), and TNF-*α* (Table 3; *r* = 0.164, *P* = 0.027).

Phe was also associated with different components of MetS, but when these associations were adjusted for age, gender, protein intake, RMR, and FI, only associations between Phe and WC (Table 3, Figure 1; *r* = 0.362, *P* = 0.006), glucose (Table 3; *r* = 0.321, *P* = 0.014), TNF-*α* (Table 3; *r* = 0.282, *P* = 0.035), and HDL-cholesterol (Table 3; *r* = −0.271, *P* = 0.038) remained significant.

### 3.6. Associations between Leu and Components of MetS and Markers of Inflammation

**Figure 2:**
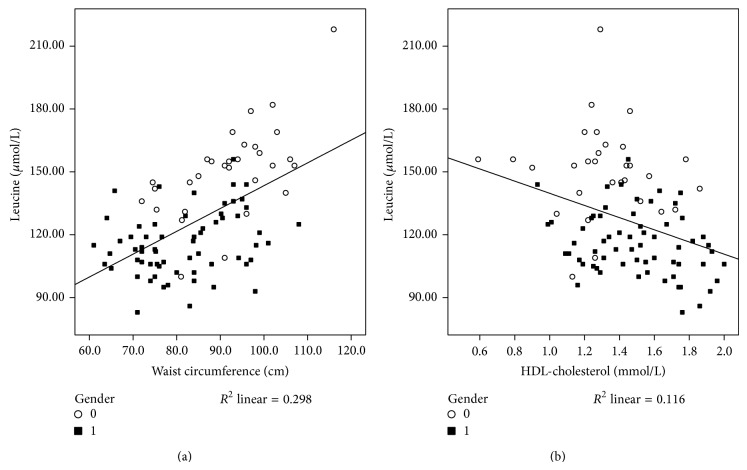
*Associations between leucine and components of MetS, waist circumference (a) and HDL-cholesterol (b)*. Evaluation of the association between leucine and components of MetS reveals a significant relationship between leucine and waist circumference (*r* = 0.546, *R* ^2^ = 0.298; *P* < 0.001) (a) and leucine and HDL-cholesterol (*r* = −0.341, *R* ^2^ = 0.116; *P* = 0.001) (b). ○, male subjects; ■, female subjects. Associations were analyzed by Pearson's correlation analyses (crude). A *P* value of less than 0.05 was taken as a statistically significant difference between the tested parameters.

Furthermore, evaluation of the association between Leu and components of MetS reveals a significant relationship between Leu and WC (Table 3, Figure 2; *r* = 0.546, *P* < 0.001), Leu and ABSI (Table 3; *r* = 0.311, *P* = 0.007), Leu and HOMA (Table 3; *r* = 0.297, *P* = 0.004), Leu and glucose (Table 3; *r* = 0.341, *P* = 0.001), Leu and triacylglycerols (Table 3; *r* = 0.223, *P* = 0.032), Leu and HDL-cholesterol (Table 3, Figure 2; *r* = −0.341, *P* = 0.001), and Leu and systolic blood pressure (SBP) (Table 3; *r* = 0.300, *P* = 0.003) but also between Leu and adiponectin (Table 3; *r* = −0.384, *P* = 0.001) and TNF-*α* (Table 3; *r* = 0.220, *P* = 0.039). When adjusting for age, gender, protein intake, RMR, and FI, we confirmed significant relationships between Leu and HOMA (Table 3; *r* = 0.351, *P* = 0.008), Leu and WC (Table 3; *r* = 0.493, *P* < 0.001), Leu and ABSI (Table 3; *r* = 0.272, *P* = 0.009), Leu and adiponectin (Table 3; *r* = −0.273, *P* = 0.038) and TNF-*α* (Table 3; *r* = 0.312, *P* = 0.012), and Leu and serum glucose (Table 3; *r* = 0.391, *P* = 0.003) and HDL-cholesterol (Table 3; *r* = −0.286, *P* = 0.026).

### 3.7. Associations between Other Amino Acids and Components of MetS and Markers of Inflammation

**Figure 3:**
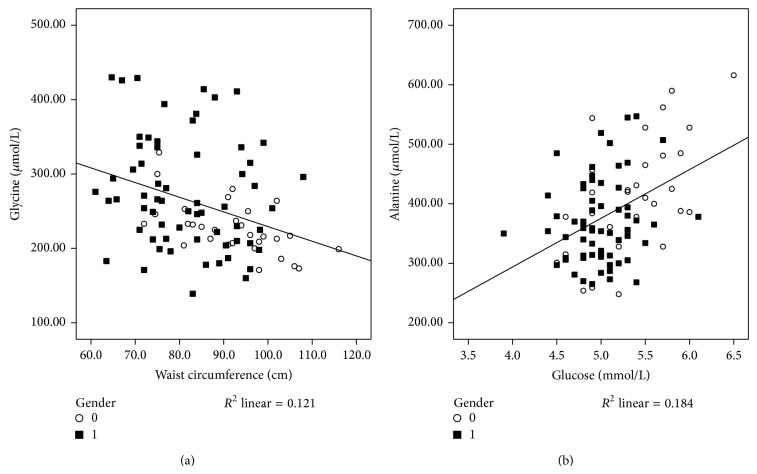
*Associations between glycine and waist circumference (a) and between alanine and glucose (b)*. A significant relationship between glycine and waist circumference is evident in (a) (*r* = −0.348, *R* ^2^ = 0.121; *P* = 0.001) and a significant relationship between alanine and glucose in (b) (*r* = 0.429, *R* ^2^ = 0.184; *P* < 0.001). ○, male subjects; ■, female subjects. Associations were analyzed by Pearson's correlation analyses (crude). A *P* value of less than 0.05 was taken as a statistically significant difference between the tested parameters.

Evaluation of the associations between Gly and components of MetS and markers of inflammation reveals significant strong negative associations between Gly and WC, SBP, triacylglycerols, CRP, and HOMA-IR, as observed in Table 3. Furthermore, even when adjusting for age, gender, protein intake, RMR, and FI, significant negative associations remained between Gly and WC (Table 3, Figure 3; *r* = −0.311, *P* = 0.016), ABSI (Table 3; *r* = −0.231, *P* = 0.025), CRP (Table 3; *r* = −0.381, *P* = 0.004), and triacylglycerols (Table 3; *r* = −0.363, *P* = 0.006).

Ala was significantly associated with SBP, visfatin, glucose (Figure 3), triacylglycerols, and HOMA-IR, and after adjusting for age, gender, protein intake, RMR, and FI, Ala remained significantly associated with visfatin, glucose, and triacylglycerols.

Hierarchical multiple regression analysis was performed to examine the effects of traditional parameters and amino acids on the level of IR score (Table 5). First the general parameters (age and gender) were entered as control variables (step 1), followed by components of MetS (WC, fasting serum concentration of glucose, HDL-cholesterol, and blood pressure) (step 2). Then specific amino acids were entered (step 3). The hierarchical multiple regression revealed that, at stage one, general parameters, for example, age and gender, contributed to the regression model and accounted for 6% of the variation in IR score. Introducing obesity parameters in stage two of the regression model explained 44% of the variation in IR score and two important predictors of IR score were confirmed (WC and fasting serum glucose). In addition, the results of the regression in step 3 indicated that serum levels of amino acids explained an additional 10% of the variation in IR score and change in *R* ^2^ was significant (*P* < 0.001). To sum up, as explained in Table 5, it was found that IR score was significantly predicted by WC (*β* = 0.565, *P* < 0.001), more than by fasting glucose (*β* = 0.382, *P* < 0.001), serum Gly (*β* = −0.188, *P* < 0.05), and serum Ala (*β* = 0.161, *P* < 0.05). Together, the adjusted *R* squared value was 0.60. This indicates that 60% of the variance in IR score was explained by the model.

## 4. Discussion

Increasingly overweight and obese population faces higher incidence of IR and related complications. IR is caused by toxic metabolic by-products that are a consequence of elevated dietary nutrients exposure of tissues, caused by obesity and overnutrition, and impaired interorgan communication networks that include hormones and cytokines [5]. As we examined the asymptomatic population, in which interventions can reverse the abrupt state, it is important to have a broad set of potential biomarkers. As Adams (2011) reports, millions of overweight to moderately obese subjects remain undiagnosed as prediabetics, as they have normal glucose levels, although they have disrupted metabolism [9]. How do the serum amino acids and adipokine relations change while progressing from the lean to obese state prior to the onset of IR? While the adipokine profiles of metabolic complications have been extensively studied, metabolic profiling is emerging as a way to get new insights into mechanism of obesity-related metabolic changes. We evaluated serum levels of 10 amino acids (Ala, Gly, Leu, Phe, Tyr, Thr, Ser, Pro, Met, and Cys) in asymptomatic adult population from Slovenia and found clear associations between a subset of amino acids and MetS characteristics. The participants were divided into three groups according to the number of components of MetS present.

When serum concentrations of the analyzed amino acids were compared, statistically significantly higher levels in MetS2 group compared to MetS0 group were observed for Cys, Tyr, Ala, Leu, and Phe while Gly and Ser levels were statistically significantly lower. Differences were observed also for CRP and TNF-*α* that were statistically significantly higher, while the difference in IL-6 was not statistically significant. Similar picture was observed when assessing adipokine signaling: adiponectin was significantly lower in MetS2 group (nonpublished data) while visfatin was significantly higher [18]. Similarly to previous reports [26, 27], we observed no difference in resistin levels between the three groups in the present study, although some studies demonstrated significantly higher serum resistin levels in MetS group compared to control group [28, 29]. Interestingly, some parameters were statistically higher already when the groups MetS0 and MetS1 were compared. These were CRP, TNF-*α*, visfatin, insulin, triacylglycerols, which have been already discussed as biomarkers, and Tyr and especially Cys. To get a better insight into the relations between serum amino acids and immune and adipose signaling, we assessed these correlations for selected amino acids, with an emphasis on Cys and Tyr.

*Cys* levels in our subjects were significantly higher with growing number of MetS components: in group MetS1, they were 10-fold higher than in group MetS0, while in group MetS2 they were 19.3 times higher than in the group MetS0. High plasma Cys showed being linked to obesity, Cys might even cause obesity and have an insulin-like action on adipocytes [30]. Total Cys showed having strong positive correlation with fat mass and being a stronger predictor of fat mass than serum lipids such as triacylglycerols, HDL, and total cholesterol [31]. We found significant positive correlations between serum Cys and HOMA-IR, CRP, TNF-*α*, WC, and ABSI and significant negative correlations between Cys and HDL-cholesterol. Serum Cys imbalances therefore correlate with markers of metabolic dysfunction. We found Cys significantly higher already in the group with only one MetS component present. The difference in Cys was the highest difference between the groups MetS0 and MetS1. Groups MetS0 and MetS1 significantly differed in conventional markers of adiposity (BMI, WC, waist/hip ratio, and body fat (%)), fitness index, and O_2_ expenditure and in levels of CRP, TNF-*α*, visfatin, insulin, and triacylglycerols, but there were no statistically significant differences in ABSI. Since ABSI is an index of both visceral obesity and decreased muscle mass [19], this could suggest that Cys levels changes are apparent before the catabolic changes in muscle (sarcopenia). Is the increased serum cysteine an early marker of a metabolic dysfunction, before the very onset of the MetS? Cys is involved in body's antioxidant defense as one of the amino acids involved in synthesis of glutathione (GSH), together with glutamate and glycine. In glucagon high states or in T2DM, Gly might become a limiting factor in GSH synthesis [32].

In the present study,* serum Gly* was lower with increasing number of components of MetS and was negatively associated with WC, serum triacylglycerols, and CRP. Although Gly is a glucogenic amino acid and the gluconeogenesis from amino acids is elevated in obese people, serum Gly concentration is lower in obese people due to their higher level of fasting insulin which is responsible for lower contribution of Gly to glucose production and higher glycogen stores [33]. Serum Gly in our participants was significantly lower in MetS2 compared to MetS0 group implying the insulin imbalance, despite the mean HOMA-IR for the latter group being below the threshold value for IR [25]. Serum Gly concentration was found as the only HOMA-IR associated predictor of both intramuscular adipose tissue (IMAT) and abdominal adiposity in functionally limited overweight older adults [34]. It negatively correlated with IMAT and positively correlated with subcutaneous adipose tissue [34]. In our study, Gly statistically negatively correlated with ABSI, a marker of visceral adiposity and decreased muscle mass, and CRP, while we found statistically significant correlation between Gly and HOMA-IR only before adjusting for age, gender, protein intake, and FI. Nevertheless, we found lower Gly as the most important predictor for both HOMA-IR and CRP in our asymptomatic younger adult population. Inflammation has an apparent role in metabolic dysfunction: high doses of salicylates or derivatives reversed IR in obese rodents [35] and also in nondiabetic insulin-resistant obese adults [36]. Growing evidence shows that Gly supplementation may be a novel therapy for obesity and T2DM. Specifically, dietary supplementation with Gly decreases concentrations of free fatty acids and triacylglycerols, as well as adipocyte size and adiposity in an animal model of intraabdominal obesity [37]. The beneficial effects of Gly in obesity and T2DM therapy can result from improved insulin sensitivity [38], increased anti-inflammatory capacity [39], and normalization of secretion of triacylglycerol-rich very low density lipoproteins from the liver by triggering neuronal transmission in the dorsal vagal complex through the *N*-methyl-*D*-aspartate receptor [40].

Sulphur-containing amino acids levels were reported to be increased in obese people, people with IR state and T2DM [9]. Although we observed substantially higher levels of Cys with growing number on MetS components, no significant association between serum levels of* Met* and components of MetS was observed in our normal and overweight asymptomatic population. However, Met is an essential amino acid whose metabolism is tightly regulated. Its metabolite, homocysteine, can be either remethylated to methionine or undergo transsulfuration to form cysteine [41]. As J. T. Brosnan and M. E. Brosnan explained, remethylation depends mainly on methionine synthase, an enzyme dependent on vitamins B2, B6, B9, and B12 [41]. The deficiency of those vitamins is associated with elevated plasma homocysteine levels. We observed no difference in homocysteine levels between the three groups in the present study. Similarly, homocysteine levels remained unchanged or were even lower in IR and T2DM without renal complications [42]. Transsulfuration, on the other hand, is increased in high Met levels, in high protein diet, and by peroxides while being decreased by antioxidants [40]. There were significant differences in protein intake between the groups. Protein intake was statistically higher in group MetS2 than in group MetS0. A trend in higher protein intake in group MetS1 was also observed, although the differences were not statistically significant. Indeed, high Cys obtained from high protein intake promotes adiposity and adverse metabolic phenotype in mice, indicating the positive association of plasma Cys with obesity in humans [43]. There was also a trend toward higher saturated fat consumption from group MetS0 to group MetS2, but the differences were not statistically significant. The differences and trends in protein and fat intake could explain the unchanged Met and homocysteine concentrations across the three groups. It could be caused by the increased rate of transsulfuration of homocysteine to Cys.

The other serum amino acid that was statistically significantly higher already in group MetS1 compared to MetS0 was* Tyr*. Its serum level was even higher in group MetS2; the difference from MetS1 was statistically significant. Tyr levels correlated with WC, ABSI, TNF-*α*, CRP, and HOMA-IR. Literature suggests a link between Tyr metabolism and insulin signaling. Tyr aminotransferase, which catalyzes the first step in degradation pathway that converts Tyr to fumarate and acetoacetate, has been well studied as a target of regulation by insulin signaling in cell models with insulin effects seen at the transcriptional and translational level [44–46]. However, a direct connection between Tyr aminotransferase and Tyr metabolism on insulin action in vertebrates has not been demonstrated. But recently Ferguson et al., based on their finding, proposed a novel role for Tyr as a developmental regulator where elevated Tyr levels play a causal role in the development of diabetes and cancer in people [47].

Also the levels of the other aromatic amino acid,* Phe*, were elevated in group MetS2 compared to MetS0. In accordance with our results, increased circulating concentrations of Tyr and Phe have often been reported in the obese, insulin-resistant, or diabetes state in humans [13, 48, 49].

However, the metabolic pathways of amino acids are interconnected, so literature reports more amino acids to be associated with IR or glucose impaired states. Branched-chain amino acids (BCAA: Val, Leu, and isoleucine (Ile)) affect the availability of aromatic amino acids by competing with them for the large neutral amino acid transporter (LAT1) [50, 51]. We also found positive associations between aromatic amino acids (Tyr and Phe) and components of MetS and insulin-resistant state. In accordance with our results, Wang et al. demonstrated that Ile, Phe, and Tyr are predictors for future diabetes with a more than fivefold higher risk [15]. In addition, it was found in a recent study that BCAA, aromatic amino acids, and orosomucoid are associated with MetS and could be relevant biomarkers of obesity associated cardiometabolic disorders [17]. Increased fasting aromatic amino acids and BCAA were reported as predictors for IR in both cross-sectional and 6-year longitudinal analysis of young normoglycaemic adults [16]. In the metabolomic study conducted by Newgard et al., principle component analysis showed that the component comprising aromatic amino acids, BCAA, and BCAA by-products were most strongly associated with obesity and positively linked to HOMA-IR [13]. How changes in Phe and Tyr metabolism could contribute to development of metabolic disease is currently unknown.

Of the BCAA, in the present study the concentration of* Leu* was determined. Leu serum concentration was statistically higher in MetS2 than in MetS0. It statistically significantly correlated with WC, ABSI, TNF-*α*, glucose, and HOMA-IR and negatively correlated with adiponectin and HDL-cholesterol (after the adjustment for age, gender, protein intake, RMR, and FI), all markers of a metabolically disrupted state. Higher serum Leu concentrations in metabolically disrupted states were reported previously [13]. Newgard and coauthors proposed that increased BCAA levels activate the mammalian target of rapamycin/protein 6 kinase 1 (mTOR/S6K1) pathway and phosphorylation of insulin receptor substrate 1 (IRS1) on multiple Ser, contributing to IR [13]. However, whether enhanced BCAA concentrations in blood serum are the reason or the cause of insulin insensitivity is not clear yet [9]. There is a lot of evidence of positive effect of Leu supplementation on metabolic health [52, 53]. However, the effects of dietary protein on metabolic health are not clear yet. In the present study, it is evident that individuals with 2 or more components of MetS consumed more protein than lean individuals in group MetS0 and association between protein intake and Leu levels in group MetS2 remained significant even after adjustment for age, gender, energy intake, and physical activity. Is higher dietary protein intake responsible for higher fasting serum Leu and observed higher fasting serum concentrations of other amino acids? Leu is an essential amino acid; thus dietary protein could have a significant impact on Leu in humans. However, as suggested, most metabolomics experiments (including the present) are performed in fasted state, so it is not likely that the observed serum amino acid levels are dependent on dietary amino acid [9]. Another potential explanation for higher fasting Leu in the present study could be impaired BCAA catabolism. In obese cotwins, decreased adipose tissue BCAA catabolism that correlated with critical clinical measures of obesity was observed compared to nonobese cotwins [54]. Further, in obese subjects, accelerated protein catabolism in muscle and muscle wasting is present due to disuse in sedentary lifestyle or due to systemic low-grade inflammation or combination of both (sarcopenic mechanisms are revised in [55]). Adams explains impaired amino acid metabolism by impaired insulin action, increased FFA oxidation, and cooccurring changes in mitochondrial redox status (which is shifted in a more reducing state) that attenuate specific catabolic pathways (blocking BCKD and PDH) that in turn increase tissue and blood concentrations of BCAA, sulphur amino acids, aromatic amino acids Phe and Tyr, and related derivatives [9].

Increased BCAA catabolic flux might contribute to increased gluconeogenesis and glucose intolerance via glutamate transamination to Ala [13]. The first step of BCAA catabolism produces Glu (due to technical restrictions we do not have the data for its concentration); the accumulation of the latter might increase the transamination of pyruvate to Ala [51]. We found statistically significantly higher serum* Ala* levels in group MetS2 than in group MetS0. Ala levels positively correlated with visfatin, glucose, and triacylglycerols after adjusting for age, gender, protein intake, RMR, and FI. In catabolic states, circulating Ala increases, acting as a carrier of amino acids from muscle to liver, where gluconeogenesis takes place. As She et al. summarized the research from four decades ago, this increase of circulating Ala can be due to transamination of glucose-derived pyruvate carrier in increased muscle catabolism or from transamination of pyruvate derived from glycolysis [56]. Could higher Ala level in group MetS2 point to a higher muscle catabolism in subjects in group MetS2? Group MetS2 had, indeed, significantly higher ABSI compared to MetS0 and MetS1 although Ala levels did not correlate with ABSI. We found Ala as important predictor of HOMA-IR. This is in accordance with a cross-sectional study of 263 nonobese Asian Indian and Chinese men, in which IR was significantly associated with the increased levels of Ala, Pro, Val, Leu/Ile, Phe, Tyr, glutamate/glutamine, and ornithine [14].

## 5. Conclusions

Overall, above cited and our results point to perturbation of amino acid metabolism in subjects with metabolic disease. However, in the present study, perturbation of Cys, Tyr, Ala, Leu, Phe, Gly, and Ser metabolism was observed in individuals with only 2 components of MetS, prior to the onset of metabolic disease. Further, Cys and Tyr were significantly higher already with one component of MetS present. We found correlations between Cys and Tyr and markers of inflammation, CRP and TNF-*α*. One of the potential mechanisms contributing to MetS is dysregulation of the adipose tissue and increased cytokine production [53]. So, our findings indicate that altered Cys and Tyr in serum not only are associated with IR, but also are closely related to inflammatory markers. Taken together, these results may indicate that altered Cys and Tyr metabolism are associated with inflammation prior to impaired glucose metabolism is observed and prior to diagnosis of MetS and therefore makes them suitable candidates for early biomarkers in asymptomatic subjects at increased risk of developing MetS.

## Figures and Tables

**Table 1:** Baseline characteristic and physiologic measures.

Variable	Group		
	MetS0 *N* = 31	MetS1 *N* = 35	MetS2 *N* = 30
Baseline characteristics			
Female	20 (65%)	24 (69%)	20 (67%)
Age (years)	36.6 ± 6.5	36.7 ± 5.6	39.5 ± 6.2
BMI (kg/m^2^)	21.6 ± 2.2	26.4 ± 4.2^b^	29.0 ± 3.5^d,e^
Waist circumference (cm)	74.1 ± 6.7	85.5 ± 9.2^b^	96.5 ± 8.4^d,f^
Waist/hip ratio	0.81 ± 0.07	0.85 ± 0.09^a^	0.90 ± 0.06^d,e^
ABSI (m^11/6^/kg^2/3^)	0.073 ± 0.005	0.074 ± 0.005	0.077 ± 0.004^d,f^
Systolic blood pres. (mmHg)	119 ± 14	122 ± 13	134 ± 20
Diastolic blood pres. (mmHg)	67 ± 6	73 ± 12	80 ± 12^c^
Pulse (rate/min)	58 ± 25	55 ± 29	66 ± 9^c,e^
Smoking (%)			
Yes	19	17	10
No	81	83	90
Dietary intake			
Total energy (kcal/day)	1950 ± 500	2050 ± 650	2100 ± 700
Total carbohydrate (g/day)	240 ± 70	250 ± 100	250 ± 100
Total fiber (g/day)	23 ± 11	21 ± 7	23 ± 10
Total protein (g/day)	75 ± 25	85 ± 30	90 ± 40^c^
Total fat (g/day)	75 ± 25	80 ± 30	80 ± 30
Total saturated fat (g/day)	23 ± 10	25 ± 10	28 ± 12
Polyunsaturated fat (g/day)	11 ± 5	11 ± 5	11 ± 6
Physical status			
Fitness index	110 ± 15	90 ± 15^a^	80 ± 15^c^
Body composition			
Body fat (%)	21.9 ± 6.6	29.9 ± 10.1^a^	35.6 ± 9.5^d,e^
Resting metabolic rate			
RMR (kcal/day)	1470 ± 300	1440 ± 300	1450 ± 400
Insulin resistance			
HOMA-IR	1.2 ± 0.5	1.4 ± 0.5	2.5 ± 1.2^d,f^

ABSI: a body shape index; BMI: body mass index; HOMA-IR: insulin resistance score; RMR: resting metabolic rate.

Values are presented as means ± SD.

^a,b^Group MetS1 is significantly different from group MetS0 at *P* < 0.05 and *P* < 0.001, respectively.

^c,d^Group MetS2 is significantly different from group MetS0 at *P* < 0.05 and *P* < 0.001, respectively.

^e,f^Group MetS2 is significantly different from group MetS1 at *P* < 0.05 and *P* < 0.001, respectively.

**Table 2:** Adipokines, cytokines, and metabolites.

Variable	MetS0 *N* = 31	MetS1 *N* = 35	MetS2 *N* = 30
Adipokines			
Adiponectin (mg/L)	6.5 ± 3.2	6.3 ± 4.6	3.7 ± 2.5^c,e^
Visfatin (*μ*g/L)	0.33 ± 0.22	0.60 ± 0.43^b^	0.96 ± 0.53^d,e^
Resistin (mg/L)	8.2 ± 2.6	8.0 ± 2.1	8.0 ± 3.4
Markers of inflammation			
C-reactive protein (mg/L)	0.7 ± 0.6	2.0 ± 1.8^b^	3.1 ± 2.1^d,e^
IL-6 (ng/L)	3.0 ± 0.8	3.0 ± 1.0	4.3 ± 1.1
TNF-*α* (ng/L)	2.0 ± 1.6	4.2 ± 2.2^a^	4.7 ± 2.1^c^
Conventional metabolites			
Glucose (mmol/L)	4.9 ± 0.4	5.1 ± 0.3	5.4 ± 0.5^c,e^
Insulin (*μ*U/mL)	5.4 ± 1.9	7.3 ± 2.8^a^	13.0 ± 5.8^d,e^
Total cholesterol (mmol/L)	4.9 ± 0.8	5.4 ± 1.0	5.6 ± 1.2^c^
HDL-cholesterol (mmol/L)	1.6 ± 0.2	1.5 ± 0.3	1.2 ± 0.3^c,e^
LDL-cholesterol (mmol/L)	3.0 ± 0.7	3.4 ± 0.9	3.6 ± 1.1^c^
Triacylglycerols (mmol/L)	0.8 ± 0.2	1.0 ± 0.4^a^	1.7 ± 0.8^d,f^
Homocysteine (*μ*mol/L)	10.8 ± 2.6	9.6 ± 2.6	10.4 ± 2.5
Bilirubin (*μ*mol/L)	14.5 ± 5.6	14.9 ± 6.5	11.9 ± 3.3^c,e^
Amino acids in serum (*μ*mol/L)			
Alanine	365 ± 72	389 ± 87	426 ± 75^c^
Glycine	274 ± 64	255 ± 73	230 ± 46^c^
Leucine	119 ± 18	124 ± 20	158 ± 30^c^
Phenylalanine	59 ± 8	63 ± 8	67 ± 6^c^
Tyrosine	54 ± 13	64 ± 11^a^	72 ± 10^d,e^
Threonine	134 ± 32	132 ± 23	131 ± 17
Serine	119 ± 17	116 ± 22	105 ± 11^c^
Proline	175 ± 63	172 ± 32	203 ± 43
Methionine	22 ± 5	23 ± 5	24 ± 3
Cysteine	0.3 ± 0.3	3.0 ± 2.7^b^	5.8 ± 4.7^d,e^

HDL: high density lipoprotein; IL: interleukin; LDL: low density lipoprotein; TNF: tumor necrosis factor.

Values are presented as means ± SD.

^a,b^Group MetS1 is significantly different from group MetS0 at *P* < 0.05 and *P* < 0.001, respectively.

^c,d^Group MetS2 is significantly different from group MetS0 at *P* < 0.05 and *P* < 0.001, respectively.

^e,f^Group MetS2 is significantly different from group MetS1 at *P* < 0.05 and *P* < 0.001, respectively.

**Table:** (a) Variables: components of MetS and markers of inflammation (r, P)^a^

AA (*μ*mol/L)	WC (cm)	ABSI (m^11/6^/kg^2/3^)	SBP (mmHg)	ADI (mg/L)	TNF-*α* (ng/L)	Visfatin (*μ*g/L)	Glc (mmol/L)	HDL-c (mmol/L)	TG (mmol/L)	CRP (mg/L)	IR
Ala	0.15 ns	0.15 ns	**0.21** *0.044 *	−0.11 ns	0.06 ns	**0.28** *0.010 *	**0.43** *0.000 *	−0.10 ns	**0.26** *0.013 *	−0.05 ns	**0.31** *0.003 *

Gly	**−0.35** *0.001 *	**−0.29** *0.015 *	**−0.26** *0.012 *	0.18 ns	−0.13 ns	0.15 Ns	−0.20 ns	0.06 ns	**−0.32** *0.002 *	**−0.28** *0.006 *	**−0.33** *0.001 *

Leu	**0.55** *0.000 *	**0.31** *0.007 *	**0.30** *0.003 *	**−0.38** *0.001 *	**0.22** *0.039 *	0.06 Ns	**0.34** *0.001 *	**−0.34** *0.001 *	**0.22** *0.032 *	0.08 ns	**0.30** *0.004 *

Cys	**0.51** *0.000 *	**0.33** *0.002 *	0.00 ns	**−0.24** *0.049 *	**0.27** *0.010 *	0.07 Ns	**0.22** *0.038 *	**−0.31** *0.002 *	0.18 ns	**0.40** *0.000 *	**0.35** *0.001 *

Phe	**0.41** *0.000 *	**0.21** *ns *	0.05 ns	−0.14 ns	**0.25** *0.018 *	−0.03 Ns	**0.25** *0.016 *	**−0.26** *0.012 *	0.08 ns	0.14 ns	**0.26** *0.011 *

Tyr	**0.62** *0.000 *	**0.32** *0.005 *	**0.23** *0.024 *	**−0.29** *0.018 *	**0.30** *0.005 *	0.13 Ns	**0.23** *0.026 *	**−0.24** *0.020 *	**0.21** *0.044 *	**0.31** *0.003 *	**0.36** *0.000 *

**Table:** (b) Correlation coefficients adjusted for age, gender, protein intake, RMR, and FI (r, P)^b^

AA (*μ*mol/L)	WC (cm)	ABSI (m^11/6^/kg^2/3^)	SBP (mmHg)	ADI (mg/L)	TNF-*α* (ng/L)	Visfatin (*μ*g/L)	Glc (mmol/L)	HDL-c (mmol/L)	TG (mmol/L)	CRP (mg/L)	IR
Ala	0.08 ns	0.13 ns	0.12 ns	−0.03 ns	0.04 ns	**0.25** *0.05 *	**0.48** *0.000 *	−0.12 ns	**0.26** *0.045 *	−0.09 ns	0.19 ns

Gly	**−0.31** *0.016 *	**−0.23** *0.025 *	−0.18 ns	0.15 ns	−0.06 ns	0.15 ns	−0.16 ns	0.16 ns	**−0.36** *0.006 *	**−0.38** *0.004 *	−0.23 ns

Leu	**0.49** *0.000 *	**0.27** *0.009 *	0.23 ns	**−0.27** *0.038 *	**0.31** *0.012 *	0.05 ns	**0.39** *0.003 *	**−0.29** *0.026 *	0.14 ns	0.26 ns	**0.35** *0.008 *

Cys	**0.51** *0.000 *	**0.32** *0.002 *	−0.06 ns	−0.20 ns	**0.26** *0.038 *	0.07 ns	0.19 ns	**−0.28** *0.032 *	0.10 ns	**0.38** *0.003 *	**0.44** *0.001 *

Phe	**0.36** *0.006 *	**0.17** *ns *	−0.08 ns	−0.18 ns	**0.28** *0.035 *	−0.21 ns	**0.32** *0.014 *	**−0.27** *0.038 *	−0.02 ns	0.16 ns	0.19 ns

Tyr	**0.50** *0.000 *	**0.27** *0.009 *	0.11 ns	−0.20 ns	**0.16** *0.027 *	0.03 ns	0.24 ns	−0.23 ns	0.11 ns	**0.29** *0.029 *	**0.31** *0.020 *

AA: amino acids in serum; ABSI: a body shape index; ADI: adiponectin; CRP: C-reactive protein; Glc: glucose; HDL-c: high density lipoprotein-cholesterol; IR: model assessment for insulin resistance; SBP: systolic blood pressure; TG: triacylglycerol; TNF: tumor necrosis factor; WC: waist circumference.

Pearson correlation coefficients, crude^a^ and adjusted for age and gender, protein intake, RMR, and FI^b^. Bold entries indicate significant correlations (*P* < 0.05).

**Table 4:** Association of cysteine and methionine with parameters of body composition^a^.

	Methionine (*μ*mol/L)	Cysteine (*μ*mol/L)
Cysteine (*μ*mol/L)	0.14^c^	—
BMI (kg/m^2^)	0.06	**0.52**
Body fat (%)	−0.05	**0.48**
Serum vitamin B12 (pg/mL)	**0.20**	0.04
Serum folic acid (*μ*g/L)	−0.09	0.19

^a^Pearson correlation coefficients, adjusted for age and gender.

Bold entries indicate significant correlations (*P* < 0.001 unless otherwise stated).

^c^ *P* < 0.05.

**Table 5:** Results of hierarchical multiple regression analysis for variables predicting insulin resistance score.

Predictors	Dependent variable: insulin resistance score		
	*β*	*F*	Δ*R* ^2^
Step 1		2.82	0.06
Age (years)	0.167		
Gender	−0.201		
Step 2		15.99	0.44^***^
Waist circumference (cm)	0.565^***^		
Systolic blood pres. (mmHg)	0.085		
Diastolic blood pres. (mmHg)	0.013		
Fasting glucose (mmol/L)	0.382^***^		
HDL-cholesterol (mmol/L)	−0.104		
Step 3		13.45	0.10^***^
Alanine (*μ*mol/L)	0.161^*^		
Glycine (*μ*mol/L)	−0.188^*^		
Leucine (*μ*mol/L)	0.020		
Tyrosine (*μ*mol/L)	0.086		
Cysteine (*μ*mol/L)	0.043		
Total *R* ^2^			0.60^***^
*N*			96

^*^ *P* < 0.05, ^**^ *P* < 0.01, and ^***^ *P* < 0.001.

## References

[1] Park Y.-W., Zhu S., Palaniappan L., Heshka S., Carnethon M. R., Heymsfield S. B. (2003). The metabolic syndrome: prevalence and associated risk factor findings in the US population from the Third National Health and Nutrition Examination Survey, 1988–1994. *Archives of Internal Medicine*.

[2] Alberti K. G. M. M., Zimmet P., Shaw J., IDF Epidemiology Task Force Consensus Group (2005). The metabolic syndrome—a new worldwide definition. *The Lancet*.

[3] Alberti K. G. M. M., Eckel R. H., Grundy S. M. (2009). Harmonizing the metabolic syndrome: a joint interim statement of the international diabetes federation task force on epidemiology and prevention; National heart, lung, and blood institute; American heart association; World heart federation; International atherosclerosis society; and international association for the study of obesity. *Circulation*.

[4] Barazzoni R., Silva V., Singer P. (2014). Clinical biomarkers in metabolic syndrome. *Nutrition in Clinical Practice*.

[5] Muoio D. M., Newgard C. B. (2008). Mechanisms of disease: molecular and metabolic mechanisms of insulin resistance and *β*-cell failure in type 2 diabetes. *Nature Reviews Molecular Cell Biology*.

[6] Ruiz-Núñez B., Pruimboom L., Dijck-Brouwer D. A. J., Muskiet F. A. J. (2013). Lifestyle and nutritional imbalances associated with Western diseases: causes and consequences of chronic systemic low-grade inflammation in an evolutionary context. *The Journal of Nutritional Biochemistry*.

[7] Munukka E., Wiklund P., Pekkala S. (2012). Women with and without metabolic disorder differ in their gut microbiota composition. *Obesity (Silver Spring)*.

[8] Wilson P. W. F., Meigs J. B., Sullivan L., Fox C. S., Nathan D. M., D’Agostino R. B. (2007). Prediction of incident diabetes mellitus in middle-aged adults: the framingham offspring study. *Archives of Internal Medicine*.

[9] Adams S. H. (2011). Emerging perspectives on essential amino acid metabolism in obesity and the insulin-resistant state. *Advances in Nutrition*.

[10] Hirabara S. M., Gorjão R., Vinolo M. A., Rodrigues A. C., Nachbar R. T., Curi R. (2012). Molecular targets related to inflammation and insulin resistance and potential interventions. *Journal of Biomedicine and Biotechnology*.

[11] Nobili V. (2012). Metabolic disorders: all that we know from circulating biomarkers. *Biomarkers in Medicine*.

[12] Huffman K. M., Shah S. H., Stevens R. D. (2009). Relationships between circulating metabolic intermediates and insulin action in overweight to obese, inactive men and women. *Diabetes Care*.

[13] Newgard C. B., An J., Bain J. R. (2009). A branched-chain amino acid-related metabolic signature that differentiates obese and lean humans and contributes to insulin resistance. *Cell Metabolism*.

[14] Tai E. S., Tan M. L. S., Stevens R. D. (2010). Insulin resistance is associated with a metabolic profile of altered protein metabolism in Chinese and Asian-Indian men. *Diabetologia*.

[15] Wang T. J., Larson M. G., Vasan R. S. (2011). Metabolite profiles and the risk of developing diabetes. *Nature Medicine*.

[16] Würtz P., Soininen P., Kangas A. J. (2013). Branched-chain and aromatic amino acidsare predictors of insulinresistance in young adults. *Diabetes Care*.

[17] Cheng S., Wiklund P. K., Pekkala S. (2014). Serum metabolic profiles in overweight and obese women with and without metabolic syndrome. *Diabetology and Metabolic Syndrome*.

[18] Jurdana M., Petelin A., Černelič Bizjak M., Biolo G., Jenko Pražnikar Z. (2013). Increased serum visfatin levels in obesity and its association with anthropometric/biochemical parameters, physical inactivity and nutrition. *e-SPEN Journal*.

[19] Biolo G., di Girolamo F. G., Breglia A. (2014). Inverse relationship between “a body shape index” (ABSI) and fat-free mass in women and men: insights into mechanisms of sarcopenic obesity. *Clinical Nutrition*.

[20] Golob T., Stibilj V., Žlender B. (2006). *Slovenian Food Composition Tables—Meat and Meat Products*.

[21] Souici S. W., Fachmann W., Kraut H. (2008). *Food Composition and Nutrition Tables*.

[22] Fitness for health: Tamper Finland. http://www.ukkinstituutti.fi/.

[23] Matthews D. R., Hosker J. P., Rudenski A. S., Naylor B. A., Treacher D. F., Turner R. C. (1985). Homeostasis model assessment: insulin resistance and *β*-cell function from fasting plasma glucose and insulin concentrations in man. *Diabetologia*.

[24] Biolo G., Agostini F., Simunic B. (2008). Positive energy balance is associated with accelerated muscle atrophy and increased erythrocyte glutathione turnover during 5 wk of bed rest. *The American Journal of Clinical Nutrition*.

[25] Stern S. E., Williams K., Ferrannini E., DeFronzo R. A., Bogardus C., Stern M. P. (2005). Identification of individuals with insulin resistance using routine clinical measurements. *Diabetes*.

[26] Lee J. H., Chan J. L., Yiannakouris N. (2003). Circulating resistin levels are not associated with obesity or insulin resistance in humans and are not regulated by fasting or leptin administration: cross-sectional and interventional studies in normal, insulin-resistant, and diabetic subjects. *The Journal of Clinical Endocrinology and Metabolism*.

[27] Kural B., Değer O., Erem C., Balaban Yücesan F., Alıyazicioğlu R., Barlak Y. (2014). Is the combined use of insulin resistance indices, including adipokines, more reliable in metabolic syndrome?. *Turkish Journal of Medical Sciences*.

[28] Asgary S., SamsamShariat S. Z., Ghorbani A., Keshvari M., Sahebkar A., Sarrafzadegan N. (2014). Relationship between serum resistin concentrations with metabolic syndrome and its components in an Iranian population. *Diabetes & Metabolic Syndrome: Clinical Research & Reviews*.

[29] Aquilante C. L., Kosmiski L. A., Knutsen S. D., Zineh I. (2008). Relationship between plasma resistin concentrations, inflammatory chemokines, and components of the metabolic syndrome in adults. *Metabolism: Clinical and Experimental*.

[30] Elshorbagy A. K., Smith A. D., Kozich V., Refsum H. (2012). Cysteine and obesity. *Obesity*.

[31] Elshorbagy A. K., Nurk E., Gjesdal C. G. (2008). Homocysteine, cysteine, and body composition in the Hordaland Homocysteine Study: does cysteine link amino acid and lipid metabolism?. *The American Journal of Clinical Nutrition*.

[32] Wu G., Fang Y.-Z., Yang S., Lupton J. R., Turner N. D. (2004). Glutathione metabolism and its implications for health. *The Journal of Nutrition*.

[33] Chevalier S., Burgess S. C., Malloy C. R., Gougeon R., Marliss E. B., Morais J. A. (2006). The greater contribution of gluconeogenesis to glucose production in obesity is related to increased whole-body protein catabolism. *Diabetes*.

[34] Lustgarten M. S., Price L. L., Phillips E. M., Fielding R. A. (2013). Serum glycine is associated with regional body fat and insulin resistance in functionally-limited older adults. *PLoS ONE*.

[35] Yuan M., Konstantopoulos N., Lee J. (2001). Reversal of obesity- and diet-induced insulin resistance with salicylates or targeted disruption of Ikk*β*. *Science*.

[36] Fernández-Real J.-M., López-Bermejo A., Ropero A.-B. (2008). Salicylates increase insulin secretion in healthy obese subjects. *The Journal of Clinical Endocrinology and Metabolism*.

[37] El Hafidi M., Pérez I., Zamora J., Soto V., Carvajal-Sandoval G., Baños G. (2004). Glycine intake decreases plasma free fatty acids, adipose cell size, and blood pressure in sucrose-fed rats. *American Journal of Physiology: Regulatory Integrative and Comparative Physiology*.

[38] Gannon M. C., Nuttall J. A., Nuttall F. Q. (2002). The metabolic response to ingested glycine. *The American Journal of Clinical Nutrition*.

[39] Sekhar R. V., Mckay S. V., Patel S. G. (2011). Glutathione synthesis is diminished in patients with uncontrolled diabetes and restored by dietary supplementation with cysteine and glycine. *Diabetes Care*.

[40] Yue J. T. Y., Mighiu P. I., Naples M., Adeli K., Lam T. K. T. (2012). Glycine normalizes hepatic triglyceride-rich VLDL secretion by triggering the CNS in high-fat fed rats. *Circulation Research*.

[41] Brosnan J. T., Brosnan M. E. (2006). The sulfur-containing amino acids: an overview. *Journal of Nutrition*.

[42] Wijekoon E. P., Brosnan M. E., Brosnan J. T. (2007). Homocysteine metabolism in diabetes. *Biochemical Society Transactions*.

[43] Elshorbagy A. K., Church C., Valdivia-Garcia M., Smith A. D., Refsum H., Cox R. (2012). Dietary cystine level affects metabolic rate and glycaemic control in adult mice. *Journal of Nutritional Biochemistry*.

[44] Gelehrter T. D., Tomkins G. M. (1970). Posttranscriptional control of tyrosine aminotransferase synthesis by insulin. *Proceedings of the National Academy of Sciences of the United States of America*.

[45] Gelehrter T. D., Emanuel J. R., Spencer C. J. (1972). Induction of tyrosine aminotransferase by dexamethasone, insulin, and serum. Characterization of the induced enzyme. *Journal of Biological Chemistry*.

[46] Messina J. L., Chatterjee A. K., Strapko H. T., Weinstock R. S. (1992). Short- and long-term effects of insulin on tyrosine aminotransferase gene expression. *Archives of Biochemistry and Biophysics*.

[47] Ferguson B. D., Liu R., Rolle C. E. (2013). The EphB4 receptor tyrosine kinase promotes lung cancer growth: a potential novel therapeutic target. *PLoS ONE*.

[48] Felig P., Marliss E., Cahill G. F. (1969). Plasma amino acid levels and insulin secretion in obesity. *The New England Journal of Medicine*.

[49] Menge B. A., Schrader H., Ritter P. R. (2010). Selective amino acid deficiency in patients with impaired glucose tolerance and type 2 diabetes. *Regulatory Peptides*.

[50] Fernstrom J. D. (2005). Branched-chain amino acids and brain function. *The Journal of Nutrition*.

[51] Newgard C. B. (2012). Interplay between lipids and branched-chain amino acids in development of insulin resistance. *Cell Metabolism*.

[52] Hirahatake K. M., Slavin J. L., Maki K. C., Adams S. H. (2014). Associations between dairy foods, diabetes, and metabolic health: potential mechanisms and future directions. *Metabolism: Clinical and Experimental*.

[53] Eller L. K., Saha D. C., Shearer J., Reimer R. A. (2013). Dietary leucine improves whole-body insulin sensitivity independent of body fat in diet-induced obese Sprague-Dawley rats. *The Journal of Nutritional Biochemistry*.

[54] Pietiläinen K. H., Naukkarinen J., Rissanen A. (2008). Global transcript profiles of fat in monozygotic twins discordant for BMI: pathways behind acquired obesity. *PLoS Medicine*.

[55] Biolo G., Cederholm T., Muscaritoli M. (2014). Muscle contractile and metabolic dysfunction is a common feature of sarcopenia of aging and chronic diseases: from sarcopenic obesity to cachexia. *Clinical Nutrition*.

[56] She P., Olson K. C., Kadota Y. (2013). Leucine and protein metabolism in obese Zucker rats. *PLoS ONE*.

